# Efficient gene editing of pig embryos by combining electroporation and lipofection

**DOI:** 10.14202/vetworld.2024.2701-2707

**Published:** 2024-11-30

**Authors:** Qingyi Lin, Nanaka Torigoe, Bin Liu, Yuichiro Nakayama, Aya Nakai, Zhao Namula, Megumi Nagahara, Fuminori Tanihara, Maki Hirata, Takeshige Otoi

**Affiliations:** 1 Bio-Innovation Research Center, Tokushima University, Tokushima, Japan; 2 Department of Animal Reproduction, Faculty of Bioscience and Bioindustry, Tokushima University, Tokushima, Japan; 3 Department of Animal Reproduction, College of Coastal Agricultural Sciences, Guangdong Ocean University, Zhanjiang, China

**Keywords:** electroporation, guide RNA sequence, lipofection, pig embryo

## Abstract

**Background and Aim:**

Mosaicism, which is characterized by the presence of wild-type and more than one mutant allele, poses a serious problem in zygotic gene modification through the clustered regularly interspaced short palindromic repeat (CRISPR)/CRISPR-associated protein 9 system. Therefore, we used pig embryos to compare the gene editing efficiencies achieved by combining electroporation and lipofection using different aminopeptidase N (APN)-targeting guide RNA (gRNA) sequences.

**Materials and Methods:**

Six gRNAs (gRNA1–6) with different target sequences were designed to target APN. *Zona pellucida* (ZP)-intact zygotes collected 10 h after the start of *in vitro* fertilization (IVF) were electroporated with each gRNA to compare their gene editing efficiency. The gRNA sequences that achieved the lowest and highest mutation rates (gRNA4 and gRNA6, respectively) were selected for additional lipofection to assess gene editing efficiency following combined treatment. As ZP removal is essential for lipofection, ZP-free zygotes were electroporated with gRNA4 or gRNA6 10 h after IVF initiation, followed by lipofection with the same gRNAs 24 or 29 h after IVF initiation. The electroporated ZP-intact and ZP-free zygotes were used as controls.

**Results:**

gRNA4 and gRNA6 exhibited the lowest and highest mutation rates, respectively. gRNA4-targeted ZP-free embryos subjected to additional lipofection 29 h after IVF initiation exhibited significantly higher total and biallelic mutation rates than ZP-intact embryos that received only electroporation. Additional lipofection of gRNA6-targeted embryos had no obvious effect on mutation rates.

**Conclusion:**

Electroporation combined with lipofection using gRNAs with low mutation rates may improve gene editing efficiency in pig embryos. However, the effects may vary based on the timing of gene editing.

## Introduction

Pigs (*Sus scrofa*) are anatomically and physio- logically similar to humans, making them important animal models for biomedical research [1]. Genetically modified pigs are beneficial for various purposes, including research on human diseases [2], xenotransplantation research [3], and animal husbandry [4]. Transmissible gastroenteritis virus (TGEV), which causes enterocyte infection and necrosis leading to dehydration, is a source of high morbidity and mortality in neonatal pigs; TGEV often causes the host to be co-infected with other porcine diarrhea-associated viruses, including porcine epidemic diarrhea virus (PEDV) and rotavirus [5]. The mortality rate of immunocompromised neonatal pigs infected with TGEV or PEDV approaches 100%, resulting in significant economic losses in the global pork industry [6]. Aminopeptidase N (APN) proteins are receptors mediating TGEV infection, and APN-knockout pigs exhibit TGEV resistance [7, 8]. Therefore, APN-modified pigs are a valuable resource for the generation of TGEV-resistant pig lines. The clustered regularly interspaced short palindromic repeat (CRISPR)/CRISPR-associated protein 9 (Cas9) system, which comprises guide RNA (gRNA) and Cas9 nuclease, is widely used for gene editing [9]. Several strategies, including microinjection, electroporation, and lipofection, have been developed for the genetic modification of pig embryos [10, 11]. Electroporation facilitates embryo gene editing by creating transient defects in the phospholipid bilayer of the cell membrane, thereby providing an alternative route for CRISPR–Cas9 complex delivery [12]. In lipofection, lipophilic reagents facilitate the introduction of foreign nucleic acids into cells, thereby increasing the cellular uptake of polynucleotides [13].

Since F1 generation is required for phenotypic analysis, mosaicism complicates such analysis. Due to the long gestation period and the time to sexual maturity of pigs, production of the F1 generation is time-consuming and expensive, thus hindering research. We previously developed and optimized electroporation and lipofection methods for delivering the CRISPR/Cas9 system to pig zygotes [10, 14]. However, because mosaicism cannot be controlled using these methods, further optimization is necessary to deliver the CRISPR/Cas9 system to pigs. The introduction of additional CRISPR/Cas9 components (such as messenger RNAs and proteins/nucleases) may improve gene editing efficiency [11]. Compared with their separate application, combining electroporation and lipofection improves gene transfer, as demonstrated in lentil protoplasts and cock spermatogonia cells [15, 16]. For pig blastocysts, we hypothesized that combining electroporation and lipofection would increase the rate of biallelic mutation by enabling the re-editing of embryos that exhibit mosaicism. However, we previously demonstrated that lipofection immediately after electroporation does not improve the total and biallelic mutation rates of myostatin in *zona pellucida* (ZP)-free zygotes [17]. The appropriate timing of CRISPR/Cas9 system introduction during embryogenesis is a key factor for efficient gene editing [18]. Moreover, we previously reported increased gene editing events in 1–8 cell stage embryos treated with lipofection at 24–34 h after IVF initiation [10]. In addition, the specific gRNA sequence highly affects gene editing efficiency. Therefore, target gene editing can be enhanced by ensuring the appropriate timing of gene editing and using efficient gRNAs.

In this study, we aimed to compare mutation efficiencies in pig embryos subjected to a combination of electroporation and lipofection using six different APN-targeting gRNA sequences (gRNAs 1–6). First, the sequences were introduced into ZP-intact zygotes through electroporation, and their gene editing efficiencies were compared. The gRNA sequences with the lowest and highest electroporation mutation rates were then selected for additional lipofection to assess their mutation efficiency following combined treatment.

## Materials and Methods

### Ethical approval

The animal experiments were approved by the Institutional Animal Care and Use Committee of Tokushima University (approval number: T28-21). Ovaries of prepubertal crossbred gilts (Landrace × Large White × Duroc) were collected from a local slaughterhouse.

### Study period and location

The data collection for this study was conducted from March to May 2023 at Bio-Innovation Research Center, Tokushima University, Japan.

### Oocyte collection, *in vitro* maturation (IVM), fertilization, and embryo culture

Oocyte collection, IVM, fertilization, and embryo culture were performed as previously described by Lin *et al*. [19]. Briefly, the ovaries of prepubertal crossbred gilts (Landrace × Large White × Duroc) were collected from a local slaughterhouse and transported in physiological saline to the laboratory at 30°C within 1 h. Cumulus–oocyte complexes (COCs) with uniformly dark and pigmented ooplasm and intact cumulus cell mass were collected from the follicles using a surgical blade. Approximately 50 COCs were cultured in 500 μL IVM medium, comprising tissue culture medium 199 with Earle’s salt (TCM 199; Thermo Fisher Scientific, Waltham, MA, USA) supplemented with 10% (v/v) porcine follicular fluid, 0.6 mM cysteine (Sigma-Aldrich, St. Louis, MO, USA), 50 μg/mL gentamicin (Sigma-Aldrich), 50 μM sodium pyruvate (Sigma-Aldrich), 50 μM β-mercaptoethanol (Wako Pure Chemical Industries, Osaka, Japan), 2 mg/mL D-sorbitol (Wako Pure Chemical Industries), 10 IU/mL equine chorionic gonadotropin (Kyoritu Seiyaku, Tokyo, Japan), and 10 IU/mL human chorionic gonadotropin (Kyoritu Seiyaku), for 22 h in a four-well dish (Nunc A/S, Roskilde, Denmark). The COCs were subsequently transferred into fresh IVM medium without hormones and cultured for 22 h at 39°C in a humidified incubator containing 5% CO_2_.

For *in vitro* fertilization (IVF), frozen-thawed spermatozoa were transferred into 5 mL porcine fertilization medium (PFM; Research Institute for the Functional Peptides Co., Yamagata, Japan) and centrifuged at 500× *g* for 5 min. Pelletized sperm were resuspended in PFM and adjusted to 1 × 10^6^ sperm/mL. Then, 50 mature oocytes were transferred into 500 μL sperm-containing PFM and co-incubated for 5 h. After co-incubation, the inseminated zygotes were denuded and cultured for 3 days in porcine zygote medium (PZM-5; Research Institute for the Functional Peptides Co.) supplemented with mineral oil (Sigma-Aldrich). The cleaved embryos were transferred to a porcine blastocyst medium (PBM; Research Institute for the Functional Peptides Co.) and cultured for 4 days. IVF and embryo culture were performed at 39°C in a humidified incubator containing 5% CO_2_, 5% O_2_, and 90% N_2_. The capacity of the embryos to develop into the blastocyst stage and the genotypes of the resulting blastocysts were then analyzed.

### Design of gRNA sequences

The Alt-R CRISPR crRNA and tracrRNA systems for gRNA were obtained from Integrated DNA Technologies (IDT, Coralville, IA, USA). The gRNA sequences were designed using the CRISPRdirect web tool (https://crispr.dbcls.jp/) [20]. To minimize off-target effects, the COSMID web tool (https://crispr.bme.gatech.edu/) [21] was used to confirm that the 14 nucleotides at the 3′ end of the designed gRNAs matched only the target regions of APN. The designed gRNAs are listed in Table-1.

**Table-1:** Guide RNA (gRNA) sequences used for sequencing analysis.

Target gene	Target sequence	PAM*	Target	Strand
gRNA 1	gcgttgtgggtaggc ggtac	CGG	Exon 2	Antisense
gRNA 2	gggtctcagcgtca cgttgt	AGG	Exon 2	Antisense
gRNA 3	atgtacaggccatcc gcgtt	GGG	Exon 2	Antisense
gRNA 4	agctcagtcctgtcg atctc	TGG	Exon 2	Antisense
gRNA 5	tctgtctgtggtgt acgccc	AGG	Exon 2	Sense
gRNA 6	cccaggtcccagag atcgac	AGG	Exon 2	Sense

* PAM=Protospacer adjacent motif

### ZP removal

ZP-free zygotes were prepared before electroporation. Briefly, zygotes were exposed to 0.5% (w/v) actinase-E (Kaken-Seiyaku Corp., Tokyo, Japan) in Dulbecco’s phosphate-buffered saline (Thermo Fisher Scientific) for 20–30 s and transferred to PZM-5 without actinase-E. After ZP removal through gentle pipetting, the ZP-free zygotes were electroporated.

### Electroporation

Electroporation was performed as previously described by Hirata *et al*. [22]. An electrode (LF501PT1-20; BEX, Tokyo, Japan) was connected to a CUY21EDIT II electroporation (BEX) and placed under a stereoscopic microscope (SMZ18; Nikon, Tokyo, Japan). Approximately 50 putative zygotes were washed with Opti-MEM I solution (Thermo Fisher Scientific), and placed in a line between the electrodes on a chamber slide filled with 10 μL of nuclease-free duplex buffer (IDT) containing 100 ng/μL of APN-targeting gRNA and 100 ng/μL of Cas9 protein (Guide-it Recombinant Cas9, Takara Bio, Shiga, Japan), and electroporated at 25 V using five pulses of 1 ms each. After electroporation, the zygotes were cultured in PZM-5 for 3 days and then in PBM for 4 days, as described above. Some zygotes were subjected to lipofection.

### Lipofectamine treatment

Lipofectamine 2000 (Thermo Fisher Scientific) was used as previously described by Lin *et al*. [19]. Briefly, lipofection solution was prepared by adding 2 μL of Lipofectamine 2000–8 μL of nuclease-free duplex buffer (IDT) containing the Cas9–gRNA ribonucleoprotein complexes, which were prepared by mixing Cas9 protein (at 300 ng/μL buffer) and gRNA (at 100 ng/μL buffer) to a final volume of 20 μL. After mixing, the solutions were incubated at 25°C for 15 min to allow the formation of CRISPR/Cas ribonucleoprotein complexes, then added to 180 μL of PZM-5 containing ZP-free zygotes was added for transfection. After transfection, the embryos were cultured individually in PZM-5 and covered with mineral oil in a 25-well dish (ART Culture Dish, Nipro, Osaka, Japan) for 3 days after IVF. Subsequently, the embryos were cultured in PBM for 4 days, as described above.

### Mutation analysis

Genomic DNA was isolated from blastocysts by boiling them in 50 mM sodium hydroxide solution (Wako Pure Chemical Industries). After neutralization, the DNA samples were subjected to polymerase chain reaction (PCR) using KOD One PCR Master Mix (Toyobo, Osaka, Japan) according to the manufacturer’s instructions with the following primers: APN, 5′-GCTCCCTTCTCACCCTCACC-3′ (forward) and 5′-CGAATCCAAGCCCTTCCTTG-3′ (reverse). PCR products were extracted through agarose gel electrophoresis using the Fast Gene Gel/PCR Extraction Kit (Nippon Genetics, Tokyo, Japan). The targeted genomic regions of the PCR products were directly sequenced through Sanger sequencing using the BigDye Terminator Cycle Sequencing Kit version 3.1 (Thermo Fisher Scientific) and an ABI 3500 Genetic Analyzer (Applied Biosystems, Foster City, CA, USA). Tracking of Indels by Decomposition was used to determine the genotype of each blastocyst [23]. The examined blastocysts were classified as biallelic mutants (no wild-type allele), mosaic mutants (carrying wild-type and more than one mutant allele), or wild-type alleles only. The mutation rate was defined as the ratio of the number of gene-edited blastocysts to the total number of sequenced blastocysts. Mutation efficiency was defined as the proportion of indel mutations in mutant blastocysts.

### Experimental design

#### Experiment 1: Confirmation of gene editing efficiency of the designed gRNAs

The gRNAs (gRNA1–6) were designed to target exon 2 of APN (Table-1). ZP-intact zygotes were collected 10 h after the IVF initiation and electroporated with gRNA and Cas9 proteins. After electroporation, the zygotes were cultured in a four-well plate for 7 days, as described above. Blastocyst formation, mutation rates, and mutation efficiency were examined in each group. Five replicate experiments were conducted.

#### Experiment 2: Gene editing efficiency after combined treatment with different APN-targeting gRNAs

An open chromatin state (such as in 2–4 cell stage embryos) improves CRISPR system access to the target site, thereby increasing mutation efficiency [24, 25]. ZP-free embryos were treated through lipofection 24 or 29 h after IVF initiation when the embryos were predominantly in the 2–4 cell stage. The gRNA sequences that achieved the lowest and highest electroporation mutation rates (gRNA4 and gRNA6, respectively) were selected for additional lipofection to assess gene editing efficiency following combined treatment. As ZP removal is essential for lipofection [17], ZP-free zygotes were prepared as described above and electroporated with gRNA4 or gRNA6 10 h after IVF initiation. After electroporation, the zygotes were cultured in PZM-5, and some were subjected to an additional 5 h of lipofection at 24 or 29 h after the start of IVF, also using gRNA4 or gRNA6. ZP-intact and ZP-free zygotes subjected to electroporation alone were used as controls. Blastocyst formation, mutation rates, and mutation efficiency were examined in each group. Five replicate experiments were conducted.

### Statistical analysis

Embryo development and mutation efficiency data were evaluated using a one-way analysis of variance followed by Fisher’s protected least significant difference test using STATVIEW (Abacus Concepts, Berkeley, CA, USA). Data were subjected to arcsine transformation before analysis. The percentage of mutated blastocysts was evaluated using the Chi-square test with Yates correction. Differences were considered significant at p < 0.05.

## Results

### Experiment 1: Confirmation of gene editing efficiency of the designed gRNAs

**Figure-1:**
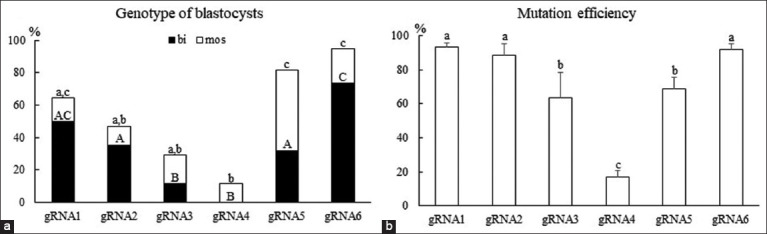
Mutations in zona pellucida (ZP)-intact zygotes edited using different aminopeptidase N (APN)-targeting guide RNA (gRNA) sequences. Six gRNAs with different target sequences were introduced into ZP-intact zygotes via electroporation 10 h after the start of *in vitro* fertilization. Five replicate experiments were conducted. (a) Blastocyst genotypes were determined using Sanger sequencing and Tracking of Indels by Decomposition analysis. Percentages were calculated by dividing the number of gene-edited blastocysts by the number of sequenced blastocysts. bi; biallelic mutant, mos; mosaic mutant. (b) Mutation efficiency in gene-edited blastocysts. Average efficiency is the proportion of indel mutations in gene-edited blastocysts. Error bars: Mean ± standard error of the mean (SEM). Groups identified by different superscripted lower or uppercase letters differ significantly (p < 0.05).

The blastocyst formation rate did not vary significantly between the ZP-intact zygotes electroporated 10 h after the start of IVF with gRNA1–6 and the non-electroporated controls (9.8%–15.9% vs. 13.8%, respectively; p > 0.05). The total and biallelic mutation rates were significantly higher in blastocysts from ZP-intact zygotes electroporated with gRNA6 (p < 0.05) than in those from zygotes electroporated with gRNA2–4 (Figure-1). Mutation efficiency was higher in blastocysts from zygotes electroporated with gRNA6 than in those with gRNA3–5. The total and biallelic mutation rates and mutation efficiency were lowest in blastocysts from zygotes electroporated with gRNA4.

### Experiment 2: Gene editing efficiency after combined treatment with different APN-targeting gRNA sequences

When gRNA4 was used, the blastocyst formation rate was similar in all groups, and the total mutation rate was significantly higher in the ZP-free embryos (electroporation alone or in combination with lipofection, p < 0.05) than in the electroporated ZP-intact embryos. Using gRNA4, mosaic and biallelic mutation rates were significantly higher in blastocysts from ZP-free embryos subjected to additional lipofection 29 h after IVF initiation (p < 0.05) than in those from electroporated ZP-intact embryos (Table-2).

**Table-2:** Development and mutation of zona pellucida (ZP)-free zygotes edited using electroporation (EP) and lipofection (LP) with gRNA4.

Group (Timing)*	Zona pellucida	No. of zygotes examined	No. (%) of embryos developed to blastocysts	No. of blastocysts examined	No. (%) of edited embryos**			

					Total	Mosaic	Biallelic	Average efficiency
EP	+	214	66 (31.1 ± 2.8)	23	2 (8.7)^a^	2 (8.7)^a^	0 (0.0)^a^	47.4
EP	–	224	70 (31.5 ± 2.5)	21	11 (52.4)^b^	9 (42.9)^b^	2 (9.5)^a,b^	40.4
EP + LP (IVF-24 h)	–	198	50 (25.1 ± 2.4)	20	8 (40.0)^b^	7 (35.0)^a,b^	1 (5.0)^a,b^	46.6
EP + LP (IVF-29 h)	–	175	53 (30.3 ± 0.8)	18	12 (66.7)^b^	7 (38.9)^b^	5 (27.8)^b^	56.6

* APN-targeting gRNA4 was introduced into zygotes through EP alone or EP followed by LP (EP+LP). EP was performed 10 h after the start of *in vitro* fertilization (IVF), and LP was performed 24 or 29 h after the start of IVF. Five replicate experiments were conducted. Percentages are expressed as the mean ± standard error of the mean (SEM).

** Percentages were calculated by dividing the number of gene-edited blastocysts by the number of examined blastocysts. Average efficiency indicates the proportion of indel mutations in mutant blastocysts, as determined through Tracking of Indels by Decomposition analysis. Total, all mutants; mosaic, mosaic mutants; biallelic, biallelic mutants. Different superscript letters in the same column indicate groups with significant differences (p < 0.05)

When gRNA6 was used, the blastocyst formation rate was significantly lower in ZP-free embryos with additional lipofection treatment (p < 0.05) than in electroporated ZP-intact embryos. Using gRNA6, the biallelic mutation rate was significantly higher in blastocysts from ZP-free embryos without lipofection (p < 0.05) than in those from electroporated ZP-intact embryos. However, lipofection did not increase the biallelic mutation rate in blastocysts. Mutation efficiency was significantly higher in blastocysts from ZP-free embryos (p < 0.05) than in those from electroporated ZP-intact embryos, irrespective of additional lipofection (Table-3).

**Table-3:** Development and mutation of zona pellucida (ZP)-free zygotes edited using electroporation (EP) and lipofection (LP) with gRNA6.

Group (Timing)*	Zona pellucida	No. of zygotes examined	No. (%) of embryos developed to blastocysts	No. of blastocysts examined	No. (%) of edited embryos**			

					Total	Mosaic	Biallelic	Average efficiency
EP	+	213	65 (30.6 ± 2.3)^a^	18	17 (94.4)	8 (44.4)^a^	9 (50.0)^a^	71.8^a^
EP	-	227	58 (25.9 ± 5.7)^a,b^	22	22 (100)	3 (13.6)^b^	19 (86.4)^b^	92.5^b^
EP + LP (IVF-24 h)	-	203	38 (18.5 ± 2.1)^b^	13	13 (100)	2 (15.4)^a,b^	11 (84.6)^a,b^	91.4^b^
EP + LP (IVF-29 h)	-	171	33 (19.2 ± 3.5)^b^	13	13 (100)	2 (15.4)^a,b^	11 (84.6)^a,b^	91.9^b^

* APN-targeting gRNA6 was introduced into zygotes via EP alone or EP followed by LP (EP+LP). EP was performed 10 h after the start of *in vitro* fertilization (IVF), and LP was performed 24 or 29 h after the start of IVF. Five replicate experiments were conducted. Percentages are expressed as the mean ± SEM.

** Percentages were calculated by dividing the number of gene-edited blastocysts by the number of examined blastocysts. Average efficiency indicates the proportion of indel mutations in mutant blastocysts determined through Tracking of Indels by Decomposition analysis. Total, all mutants; mosaic, mosaic mutants; biallelic, biallelic mutants. Different superscript letters in the same column indicate groups with significant differences (p < 0.05)

## Discussion

This study investigated whether combined electroporation and lipofection improves pig zygote gene editing. Using electroporation, we first evaluated the effects of the APN-targeting gRNAs; differences among these gRNAs did not affect the blastocyst formation rate, but did affect their mutation efficiency. The number, position, and distribution of nucleotide mismatches between single gRNA and genomic DNA are important parameters that affect mutagenesis [26]. Moreover, gene editing efficiency is affected by CRISPR/Cas9 stability because RNA is susceptible to enzymatic degradation [27]. Re-editing may improve gene editing efficiency by facilitating the repeated introduction of the CRISPR/Cas9 system and maximizing CRISPR/Cas9 stability during gene editing. We found that double electroporation using ZP-intact zygotes increased the total mutation rate, although this increase varied among target genes (unpublished data). Therefore, we selected lipofection as the re-editing method in this study to evaluate whether this combination improves gene editing efficiency.

Lipofectamine is cytotoxic, inducing ca. 35%–65% cell death under certain conditions [28]. Using gRNA4, the blastocyst formation rate of ZP-free zygotes following additional lipofection was similar to that of ZP-intact and ZP-free zygotes following electroporation alone. Using gRNA6, the blastocyst formation rate was lower in lipofection-treated ZP-free zygotes than in electroporated ZP-intact zygotes. These results indicate that the effect of additional lipofection on embryonic development depends on the gRNA sequence used, which is consistent with prior findings [29].

Using gRNA4 (which achieved the lowest mutation rate), the ZP-free zygotes exhibited higher total mutation rates than the ZP-intact zygotes in both the separate and combined treatments. This result is consistent with our previous finding that increasing zygote permeability by removing ZP, which facilitates efficient CRISPR introduction, significantly increased the total blastocyst mutation rate [30]. It has been suggested that genome editing through lipofection is possible with ZP using denuded oocytes during IVF [31]. However, lipofection of oocytes is toxic under certain conditions, as evidenced by the low cleavage and blastocyst formation rates observed [31, 32]. We previously demonstrated that ZP removal is required for CRISPR/Cas9-based genome editing in zygotes through lipofection and that it increases the mutation rates in the resulting blastocysts [17]. Moreover, culturing embryos individually in 25-well dishes significantly improved the development of ZP-free embryos, thereby minimizing the damage to embryos caused by ZP removal [33]. In this study, although additional lipofection using gRNA4 did not improve the total mutation rate, the biallelic mutation rate was significantly higher following gRNA4-mediated lipofection of ZP-free embryos at 29 h after IVF initiation than in ZP-intact embryos without lipofection. The introduction of the CRISPR/Cas9 system closer to pronucleus formation enhances mRNA translation, thereby increasing the biallelic mutation rate [34]. In this study, although electroporation was performed approximately 10 h after the start of IVF, within the time window of the onset of pronuclear formation, no biallelic mutations were observed in the ZP-intact embryos generated using gRNA4 without lipofection. Mosaicism can occur when electroporated zygotes enter the genome replication stage and undergo their first cell division, resulting in more than one cell type with different mutations [35]. We previously reported increased gene editing events in 1–8 cell stage embryos treated with lipofection at 29 h after IVF initiation [10]. The biallelic mutation rate was significantly higher after additional lipofection with gRNA4, which achieved the lowest electroporation mutation rate. These findings indicate that additional lipofection using gRNA4 improves the potential for re-editing embryos exhibiting mosaicism by applying different gene-editing machinery. In contrast, additional lipofection using gRNA6, which achieved the highest mutation rate following lipofection, had no obvious effect on the biallelic mutation rate.

These findings improved pig embryo gene editing efficiency by combining electroporation and lipofection using APN-targeting gRNA, which generates a low mutation rate. However, it is worth considering whether additional lipofection improves gene editing efficiency. Clarifying this requires a better understanding of the mechanisms of re-editing. Therefore, to elucidate the effects of lipofection in re-editing, it may be necessary to distinguish between primary and secondary editing processes using synthetic probes (i.e., conjugates of oligonucleotides and fluorophores) to visualize target RNA [36].

## Conclusion

Although lipofection following electroporation did not improve the gene editing efficiency of highly mutated APN, it increased the biallelic mutation rate of APN exhibiting a low mutation rate. These findings suggest that electroporation followed by lipofection using gRNA, which generates a low mutation rate, improves pig embryo gene editing efficiency. Nonetheless, the mechanisms underlying the lipofection-mediated increase in editing efficiency remain unclear, and this process requires validation.

## Data Availability

The data generated and/or analyzed in this study are available from the corresponding author on reasonable request.

## Authors’ Contributions

QL and TO: Conceived the study and drafted the manuscript. QL: Performed the experiments. TO: Designed the study, supervised the experiments, and reviewed the manuscript. MH: Performed genotypic analysis. NT, BL, YN, AN, and ZN: Conducted the laboratory work and statistical analyses. MN and FT: Conducted the laboratory work and revised the manuscript. All authors have read and approved the manuscript.
